# Core transcriptional regulatory circuits in prion diseases

**DOI:** 10.1186/s13041-020-0551-3

**Published:** 2020-01-20

**Authors:** Taek-Kyun Kim, Inyoul Lee, Ji-Hoon Cho, Brenda Canine, Andrew Keller, Nathan D. Price, Daehee Hwang, George Carlson, Leroy Hood

**Affiliations:** 1 Institute for Systems Biology, Seattle, WA 98109 USA; 2 Department of Structural Biology and Developmental Neurobiology, Center for Proteomics and Metabolomics, St. Jude Children’s Research Hospital, Memphis, TN 38105 USA; 3 McLaughlin Research Institute, Great Falls, MT 59405 USA; 4 Center for Plant Aging Research, Institute for Basic Science, Daegu Gyeongbuk Institute of Science and Technology, Daegu, 42988 Republic of Korea; 5 Department of Biological Sciences, Seoul National University, Seoul, 08826 Republic of Korea; 6 Institute for Neurodegenerative Diseases, Weill Institute for Neurosciences, University of California, San Francisco, CA 94158 USA

**Keywords:** Murine PrP-prion disease, Gene expression, Transcriptional regulatory circuits, Feed-forward loops, Brain cell type

## Abstract

Complex diseases involve dynamic perturbations of pathophysiological processes during disease progression. Transcriptional programs underlying such perturbations are unknown in many diseases. Here, we present core transcriptional regulatory circuits underlying early and late perturbations in prion disease. We first identified cellular processes perturbed early and late using time-course gene expression data from three prion-infected mouse strains. We then built a transcriptional regulatory network (TRN) describing regulation of early and late processes. We found over-represented feed-forward loops (FFLs) comprising transcription factor (TF) pairs and target genes in the TRN. Using gene expression data of brain cell types, we further selected active FFLs where TF pairs and target genes were expressed in the same cell type and showed correlated temporal expression changes in the brain. We finally determined core transcriptional regulatory circuits by combining these active FFLs. These circuits provide insights into transcriptional programs for early and late pathophysiological processes in prion disease.

## Introduction

Living organisms execute diverse cellular processes by operation of biological networks [1]. Such operation of the networks is perturbed under pathological conditions, involving changes of nodes in their abundances and/or edges in their activities. These changes result in dynamic perturbations of pathophysiological processes during the course of disease progression [2]. Biological networks include protein-protein interaction (PPI) [2], transcriptional regulatory networks (TRNs) [3], and metabolic networks [4]. TRNs delineate the regulations of target genes associated with cellular processes by transcriptional factors (TFs). TRNs are composed of regulatory motifs, such as transcriptional feedback and feed-forward loops [5, 6]. Expression changes of TFs and target genes included in the regulatory motifs along disease progression can represent perturbations of early- and late-stage disease processes. However, the regulatory motifs underlying these disease-perturbed processes have rarely been studied longitudinally through both early and late stages.

Transmissible PrP-prion disease, which is caused by misfolding, aggregation, and spread of misfolded forms of prion protein (PrP^Sc^), is an excellent model system to study dynamic perturbations of pathophysiological processes during disease progression since disease initiation is defined by inoculation. Prions are proteins that self-replicate through templated misfolding, accumulation, and aggregation, often, but not always, accompanied by formation of amyloid [7]. Some prions cause neurodegenerative diseases [8–11]. The prototypical prion diseases, which include scrapie in sheep and Creutzfeldt-Jakob Disease (CJD) in humans, are caused by misfolding of normal isoforms of prion protein (PrP^C^) to malignant disease-causing isoforms (PrP^Sc^). As a group, neurodegenerative diseases involving PrP are designated as PrP-prion diseases that can be transmitted experimentally as are the mouse prions used in this study, or iatrogenically, as exemplified by transmission of disease by contaminating CJD-PrP^Sc^ in surgical procedures, transplantation, or injection of cadaver-derived human pituitary growth hormone [12, 13].

Additional neurodegenerative diseases share the processes that are the defining features of prion disorders– self-replication of specific proteins through templated changes in conformation, aggregation, spread from cell-to-cell within the brain, and the ability of protein aggregates to specifically infect cultured cells and transgenic mice [8, 9]. The α-synucleinopathy multiple system atrophy (MSA) has been transmitted to mice expressing human α-synuclein transgenes by inoculation of brain homogenates from deceased patients [14]. MSA prions also infected cultured reporter cells [15]. Similarly, tau prions from Alzheimer’s disease (AD) and chronic traumatic encephalopathy propagated in cultured cells [16], and pathological tau from AD or corticobasal degeneration brains induced spreading tauopathy in tau transgenic mice [17]. Aβ prions also propagated in mice and cultured cells, maintaining conformationally determined strain properties [18]. Strikingly, the possibility of human-to-human transmission of AD pathology is supported by the presence of Aβ pathology at an early age in some patients that received cadaveric pituitary growth hormone [19].

Inoculation of prions defines the start of the disease process. Following inoculation, when prion replication is initiated in the host, there is a long interval when prions accumulate with no obvious clinical signs. The clinical phase is relatively short, though pathological changes are occurring. The pathological features over the interval between inoculation, illness, and death comprise prion replication and accumulation [20], microglia and astrocyte activation [21, 22], synaptic degeneration [23], and neuronal cell death [24]. Through comprehensive time-course gene expression analyses of eight mouse strains infected with one of two distinct prion strains (RML and 301 V), we previously showed dynamic perturbations of cellular processes associated with these four pathological features along disease progression at the whole brain level [2]. In the early stage, the complement system and leukocyte infiltration associated with microglia and astrocyte activation are the first response to PrP^Sc^ misfolding and aggregation. In the middle stages of PrP^Sc^ accumulation, glycosaminoglycans, cholesterol, and sphingolipid metabolisms become activated. Finally, in the late stage of disease, synaptic transmission and axon guidance expressions of genes associated with synaptic degeneration are down-regulated, followed by activation of cellular processes associated with neuronal cell death.

Several network models have been developed based on our time-course gene expression profiles of eight prion-mouse strain combinations. First, we provided dynamic PPI network models describing temporal perturbations of cellular processes associated with the aforementioned four groups of pathological features. Second, for each prion-mouse strain combination, Newaz et al. developed protein function network (PFN) models describing functional associations among differentially expressed genes (DEGs) at individual time points and then proposed PI3K-AKT signaling pathway as a core regulatory module through the analysis of the PFNs [25]. Third, Crespo et al. provided a gene regulatory network model and then identified a core module for regulation of prion replication and accumulation and neuronal cell death [26]. Despite the gene regulatory network, a TRN describing the regulation of early and late pathological features by TFs is lacking. Here, we present core transcriptional regulatory circuits that represent early and late perturbations of cellular processes along prion disease progression, providing insights into transcriptional programs for early and late pathophysiological processes in prion-infected brains. The significance of our findings may extend beyond PrP-prion disease to other, more common neurodegenerative diseases that share prion-related mechanisms, though the results presented here were all obtained from PrP-prion diseases, which in this paper are subsequently referred to as ‘prion diseases’.

## Materials and methods

### Identification of major differential expression patterns

**Fig. 1:**
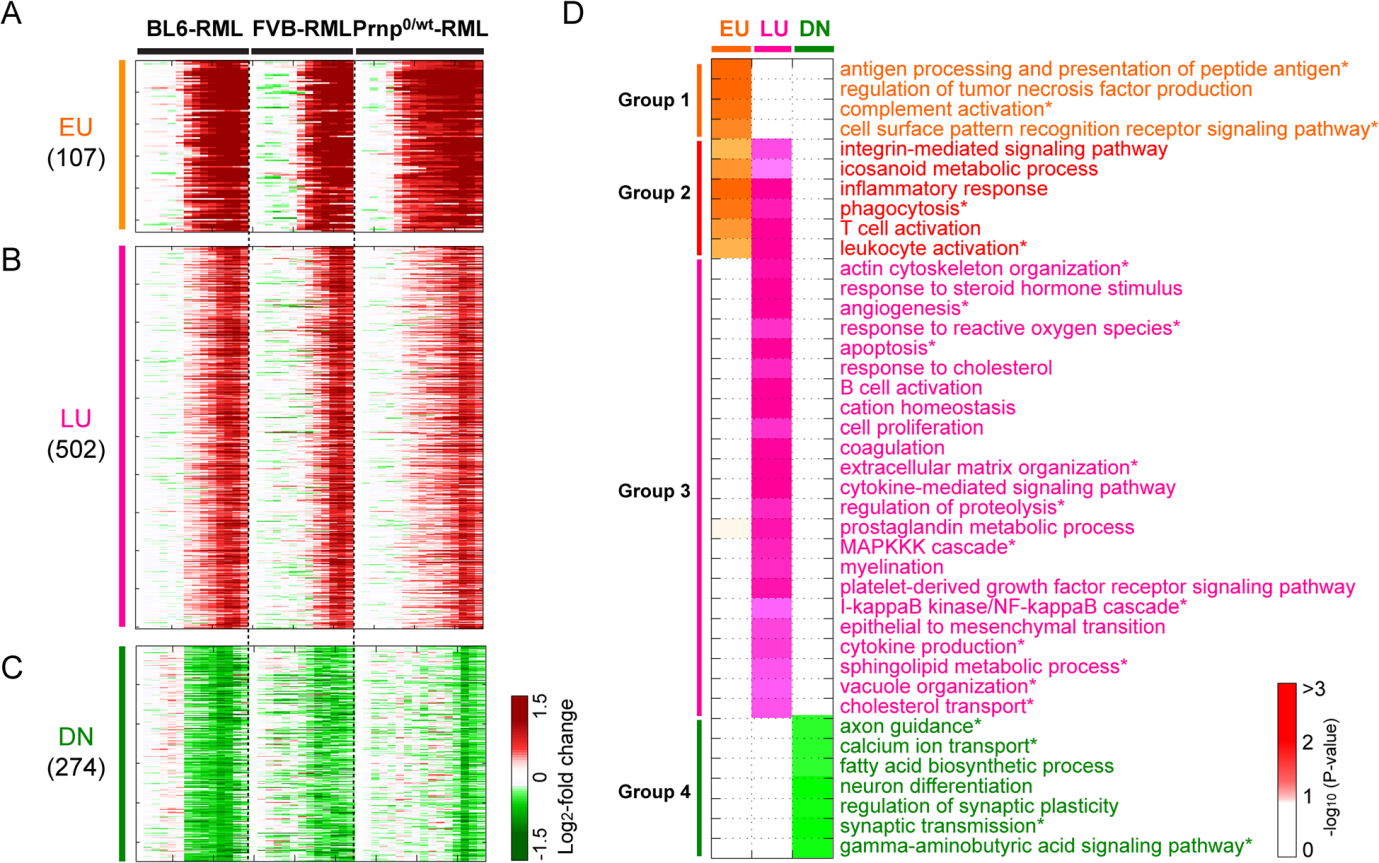
Early and late alterations of cellular processes associated with prion diseases along disease progression. **A-C**) Three major differential expression patterns shared in B6-RML, FVB-RML, and Prnp0/+ − RML along disease progression. These patterns included 107 early up-regulated (EU; **A**), 502 late up-regulated (LU; **B**), and 274 down-regulated genes (DN; **C**). Gene expression changes were shown as log_2_-fold-changes between prion-infected and control brains at individual time points. Red and green colors indicate up- and down-regulation (positive and negative log_2_-fold-changes; see the color bar), respectively. **D**) Gene ontology biological processes (GOBPs) enriched by the genes in each expression pattern. The color bar indicates the gradient of the enrichment *p*-value obtained from the DAVID software. Different colors were used to distinguish the enrichment for EU (orange), LU (magenta), and DN patterns (green). GOBPs were categorized into four (Groups 1 to 4) based on the enrichment patterns in the EU, LU, and DN gene clusters

To identify the genes showing shared dynamic expression patterns in RML infected B6, FVB, and Prnp0/wt, we first computed log_2_-fold-changes between prion-infected and control samples in each of the three mouse-prion strain combinations (B6-RML, FVB-RML, and Prnp0/wt-RML). After combining the three sets of log_2_-fold-changes into a fold change matrix, we then applied the orthogonal non-negative matrix factorization (ONMF)-based clustering method [27] to the fold change matrix. To prevent the NMF result from being biased toward the combination with large fold-changes, each of the matrices in each mouse-prion combination was converted into a vector and then the vectors from the three mouse-prion combinations were normalized by the quantile normalization method [28]. Finally, the normalized vectors were reconstructed into log_2_-fold change matrixes for the three mouse-prion combinations. NMF was then performed using the reconstructed log_2_-fold change matrixes with the following parameters: C = 1, T = 0.5, K = 5, the number of bases = 20, the number of iterations in each NMF application = 1000 and the number of NMF applications = 30. The resulting 30 differential expression patterns were categorized into the following three groups (Additional file 1: Figure S1): 1) three patterns shared across all the three combinations (Clusters 1, 2, and 3); 2) five patterns shared between two of the three combinations (Clusters 4, 6, 7, 8, and 20); and 3) the remaining 12 patterns observed only in one of the three combinations. To focus on the patterns shared across the three combinations, we selected Clusters 1, 2, and 3 representing late (LU) and early up-regulation (EU) and down-regulation (DN), respectively, and then identified the genes whose expression is significantly (*P* < 0.01) correlated with the selected patterns [27]. To remove potential false positives, we further selected the genes whose maximum absolute log_2_-fold-changes are larger than a cutoff value at least in one of the three combinations. The cutoff value was determined as the 95th percentile of the log_2_-fold-changes. Using this method, 107 (Cluster 1), 502 (Cluster 2), and 274 genes (Cluster 3) were finally identified as EU (Fig. 1a), LU (Fig. 1b), and DN (Fig. 1c) genes, respectively.

### Identification of cellular functions represented by EU, LU, and DN genes

We performed enrichment analyses of GOBPs for 107 EU, 502 LU, and 274 DN genes using The Database for Annotation, Visualization and Integrated Discovery (DAVID, ver 6.7) software [29]. Among the resulting GOBPs, we selected the GOBPs with *P* < 0.05 and count > = 3 as those enriched by EU, LU, and DN genes (Tables S2). When selecting GO terms related to the three prion disease pathophysiological features (Figs. 1d-e), for multiple redundant GO terms representing a cellular event, we selected only the most representative ones assigned to the largest numbers of genes in each pattern.

### Analysis of TF-target gene data

We obtained 7489 TF-target interactions for 748 of the 883 genes from MetaCore™ (ver 6.7 [30]; and Ingenuity Pathway Analysis (IPA) (Additional file 2: Table S3). We used ‘Direct interactions’ and ‘Transcription regulation’ options in MetaCore and ‘Upstream Regulator’ of ‘Core analysis’ in IPA. For each TF, we obtained the number of targets using the TF-target interactions and then computed statistical significance for the target number based on the following random sampling experiments: 1) 883 genes were randomly selected from the genome; 2) the number of targets for the TF was obtained; 3) Steps 1–2 were repeated 10,000 times; 4) an empirical distribution was estimated using Gaussian kernel density estimator (Bowman. 1997); and 5) an FDR for the observed target number was calculated using the empirical distribution as previously described [31]. Finally, we selected 114 major TFs with FDR < 0.1.

### Reconstruction of the TRN

**Fig. 2:**
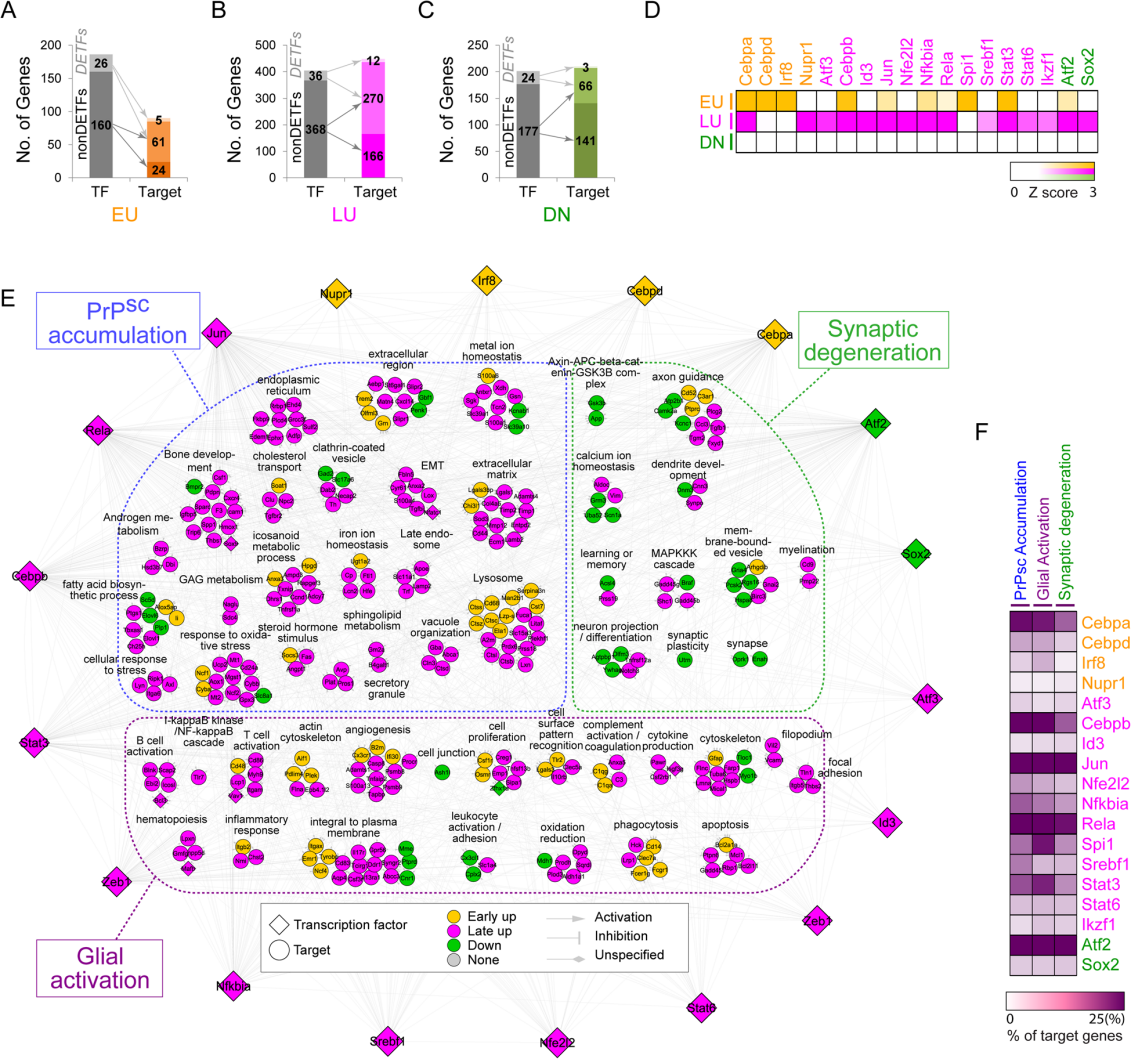
TRN describing transcriptional regulation of cellular processes associated with prion diseases. **A-C**) Early (EU; **A**) and late up-regulated (LU; **B**) and down-regulated genes (DN; **C**) regulated by the TFs. In the stacked bars, the dark and light shaded areas of the target genes represent the portions of the EU, LU, and DN genes regulated by the differentially expressed TFs (DETFs) and non-DETFs, respectively, whereas the mid-color shaded areas represent the portions of the genes regulated by both DETFs and non-DETFs (see the arrows). Different colors were used to distinguish the TF-target regulatory relationships for EU (orange), LU (magenta), and DN patterns (green). The numbers regulated by the TFs are denoted. **D**) Percentages of the target genes regulated by the 18 key TFs in each group of 107 EU, 502 LU, and 274 DN genes. The color bar represents the gradient of Z-score reflecting the percentage of the target genes regulated by each TF (Materials and Methods). **E**) A TRN describing regulations of 18 key TFs with their targets. The TRN is categorized into three transcriptional regulatory subnetworks associated with PrP^Sc^ accumulation, microglial/astrocytic activation, and synaptic degeneration, respectively. The 18 key TFs are arranged in a circle surrounding the three subnetworks. Node shapes denote TFs (diamonds) and target genes (circles); node colors represent EU (orange), LU (purple), and DN (green) nodes; and edge types indicate activation (arrow), inhibition, and unspecified interactions (blunted arrow). **F**) Percentages of the target genes regulated by the 18 key TFs in the three subnetworks. The color bar represents the gradient of the percentage of the target genes regulated by each TF

We generated a TRN for the TFs and their targets using 7489 TF-target interactions obtained as described in the previous section. In the TRN, we arranged the nodes such that the nodes with the same GOBP were grouped into the GOBP module. We then decomposed the TRN into three subnetworks (PrP accumulation, microglial/astrocytic activation, and synaptic degeneration), based on the associations of the GOBP with the three pathophysiological features (Fig. 2b and Additional file 1: Figure S2B). The nodes with no TF-target interaction available were located to the modules with the same GOBPs while the nodes with no assigned GOBP available were located to the modules functionally closest to them, based on their functional information obtained from prior literature.

### Identification of over-represented regulatory motifs

To assess characteristics of scale-free and hierarchical networks of two TRNs for all 467 TFs and 114 major TFs, respectively, for each node, we computed in- and out-degrees (*k*) and clustering coefficient *C* as previously described [32]. We then performed log-log scatter plot analyses of in- or out-degree vs. the degree distribution *P*(*k*) and degree vs. the clustering coefficient distribution *C*(*k*) (Additional file 1: Figures S3A-B). The analyses revealed negative linear relationships on the log-log scatter plots, suggesting that the TRN is scale-free and hierarchical. Next, to identify the over-represented regulatory structures between the TFs and their target genes, we performed enrichment analyses on 13 previously reported motifs using mfinder software [33]. Briefly, for Motif *i*, we generated 1000 randomized TRNs from the real TRN by randomly rewiring the edges in the TRN. The Z-score (*Z*_*i*_) was then computed: ${Z}_i=\frac{N_i^{real}-\mathrm{mean}\left({N}_i^{rand}\right)}{\mathrm{std}\left({N}_i^{rand}\right)}$ where ${N}_i^{real}$ is the frequency of Motif *i* in the real TRN, and mean(${N}_i^{rand}$) and std.(${N}_i^{rand}$) represent the mean and standard deviation of the frequencies observed in the 1000 randomized TRNs. Finally, the Z-score was then normalized to estimate its relative significance by the norm of the Z-score vector (Milo et al., 2004): ${\overset{\sim }{Z}}_i=\frac{Z_i}{{\left(\sum {Z}_i^2\right)}^{1/2}}$.

### Association of targets for TF pairs with DEGs involved in prion disease-related processes

For the overlapping *m* targets for a TF pair with *n* genes involved in a cellular process associated with prion disease, we estimated the significance of the overlapping targets using the following hypergeometric test: $$ P=1-\sum \limits_{i=0}^{m-1}\frac{\left(\begin{array}{c}n\\ {}i\end{array}\right)\left(\begin{array}{c}N-n\\ {}M-i\end{array}\right)}{\left(\begin{array}{c}N\\ {}M\end{array}\right)} $$.

### Regulation of GOBPs by 18 key TFs

To identify the relationships between 18 key TFs and 40 GOBPs enriched by the 883 EU, LU, or DN genes, the number of target genes regulated by each of the 18 key TFs among the EU, LU, or DN genes involved in each GOBP was counted. When the number of target genes is less than 2, the percentages were set to zero to remove false interpretation arising from high percentages due to the residual number of target genes.

## Results

### Dynamic perturbations of cellular processes associated with prion diseases

Previously, we proposed core genes associated with pathogenesis of prion disease that showed shared dynamic changes in differential expression along progression of prion disease [2]. To identify these core genes, we first selected the following three combinations among the eight prion strain-mouse strain combinations previously reported [2]: 1) inbred C57BL/6 J (B6) and 2) FVB/NCr (FVB) mouse strains infected with RML (B6-RML and FVB-RML), and 3) FVB background mice expressing half of the amount of PrP^C^ (*Prnp*^*0/wt*^) as wild type mice infected with RML (Prnp0/wt-RML). Prnp0/wt-RML mice have a longer incubation times (more than 250 days) than B6- and FVB-RML (~ 150 days) and a slower rate of synaptic degeneration than B6- and FVB-RML, despite a rate of PrP^Sc^ accumulation similar to that of FVB-RML. The inclusion of Prnp0/wt-RML enabled us to reliably discern early and late responsive genes.

Using the time-course gene expression profiles of the three selected prion-mouse strain combinations (B6-RML, FVB-RML, and Prnp0/wt-RML), we performed the orthogonal non-negative matrix factorization (ONMF) clustering [34] with the number of clusters = 20. Among the 20 clusters (Additional file 1: Figure S1), we focused on three clusters (Clusters 1–3) including 883 differentially expressed genes (DEGs) that showed the following shared differential expression patterns across the three combinations: 107 early up-regulated genes in Cluster 1 (Fig. 1a, EU); 502 late up-regulated genes in Cluster 2 (Fig. 1b, LU); and 274 down-regulated genes at the late stage in Cluster 3 (Fig. 1c, DN). Many of these 833 DEGs have been identified previously as DEGs in prion-infected mice (Additional file 2: Table S1) [35–42]. Moreover, of the 883 DEGs, 270 and 541 overlapped with the previous 333 core genes and 923 prion disease-related genes identified from five prion-mouse strain combinations (B6-RML, FVB-RML, C57BL/6.I-1(B6.I)-RML, B6-301V, and B6.I-301 V), respectively (Additional file 2: Table S1).

To examine how these genes represent early and late perturbations of cellular processes associated with prion diseases, we performed enrichment analyses of gene ontology biological processes (GOBPs) for EU, LU, and DN genes (Fig. 1d; Additional file 2: Table S2) using DAVID software [29]. The GOBPs represented by each cluster of genes reflect cellular processes perturbed with the corresponding dynamics. Early activated GOBPs (Groups 1 and 2 in Fig. 1d) include the processes related to innate immune or inflammatory responses (e.g. antigen processing and presentation, complement activation, pattern recognition receptor signaling pathway, phagocytosis, and leukocyte activation). Late activated GOBPs (Group 3 in Fig. 1d) include: 1) innate and/or adaptive immune responses (e.g. cytokine production, cytokine-mediated signaling, NFKB/MAPK signaling, and B-cell activation); 2) PrP^Sc^ deposition and transport (vacuole organization, regulation of proteolysis, extracellular matrix organization, and actin cytoskeleton organization); 3) lipid metabolism and transport (sphingolipid metabolism and cholesterol transport); and 4) stress responses (angiogenesis, responses to reactive oxygen species, and apoptosis). Finally, down-regulated GOBPs (Group 4 in Fig. 1d) include synaptic degeneration-related processes (axon guidance, calcium ion transport, synaptic transmission, and gamma-aminobutyric acid signaling). Early and late perturbations of these processes were consistent with those in the previous PPI network models [2].

### TRN describing transcriptional regulation of early and late perturbed processes associated with prion diseases

To investigate transcriptional regulation underlying early and late perturbations of cellular processes associated with prion diseases, we built TRN describing regulations for EU, LU, and DN genes by 467 TFs (Additional file 1: Figure S2A) based on TF-target interactions in MetaCore™ (ver 6.7) and Ingenuity Pathway Analysis (IPA). Based on these TF-target interactions, the upstream TFs were available for 84.7% (748 of 883) of the EU, LU, DN genes. The 467 TFs in the TRN were first categorized into two groups based on their differential expression: 1) 40 differentially expressed TFs (DETFs) including 5 EU, 24 LU, and 11 DN TFs and 2) 427 non-differentially expressed TFs (non-DETFs). Of the 107 EU genes, 90 (84.1%) can be transcriptionally regulated by 26 DETFs and 160 non-DETFs (Fig. 2a). Of the 502 LU genes, 448 (89.2%) can be regulated by 36 DEFTs and by 368 non-DETFs (Fig. 2b). Finally, of the 274 DN genes, 210 (76.6%) can be regulated by 24 DETFs and 177 non-DETFs (Fig. 2c). Interestingly, the DETFs can regulate larger numbers of the target genes per TF compared to the non-DETFs (Figs. 2a-c). For example, 66 EU genes were regulated by 26 DETFs (66/26 = 2.54), while 85 EU genes were regulated by 160 non-DETFs (85/160 = 0.53) (Fig. 2a). These data indicate greater relative importance of the DETFs in transcriptional regulation of the EU, LU, and DN genes.

Among the 467 TFs, we next selected 114 major TFs that have significant (FDR < 0.1) numbers of target genes in EU, LU, or DN clusters (Additional file 2: Table S3). Based on the observation that DETFs have larger numbers of targets than non-DETFs, among the 114 major TFs, we selected the following 18 DETFs as key TFs that play important roles in transcriptional regulation of cellular processes represented by the EU, LU, and DN genes: 1) 4 EU-DETFs (Cebpa/d, Irf8, and Nupr1); 2) 12 LU-DETFs (Atf3, Cebpb, Jun, Nfe2l2, Rela, Spi1, Srebf1, Stat3/6, Ikzf1, Nfkbia, and Id3); and 3) 2 DN-DETFs (Atf2 and Sox2). Interestingly, these 18 key TFs, including two down-regulated TFs, were found to regulate mostly the EU and LU genes, according to the TF-target interactome data used (Fig. 2d). Several of the 18 key TFs have been reported to have potential involvement in prion diseases or other neurodegenerative diseases (Additional file 2: Table S3). For example, Stat3 showed increased phosphorylation in mouse brains infected with prions, suggesting its potential roles in pathogenesis of prion disease [43]. Also, Atf2, an abundant TF in normal brains, was significantly down-regulated in the brains with Alzheimer’s, Parkinson’s, and Huntington’s diseases, consistent with our findings [44]. Moreover, a dominant negative TF Jun significantly reduced neuronal death in prion infected neurons [45], and Nfe2l2, also known as Nrf2, was strongly up-regulated in multiple sclerosis lesions and found to be associated with active demyelination in the lesions [46].

We then developed a TRN model using the 18 key TFs and 315 target genes (42.1% of 748 DEGs) based on the TF-target interactome data (Fig. 2e). The target genes in the TRN were organized into three subnetworks that represent three aspects of disease: PrP^Sc^ accumulation, microglial/astrocytic activation, and synaptic degeneration [2]. We then examined how significantly the 18 key TFs regulate cellular processes associated with the three pathological features (Fig. 1d) by computing the fraction of their target genes to the DEGs involved in each cellular process (Additional file 1: Figure S2B; Additional file 2: Table S4). For example, the processes represented only by the EU genes (Group 1 in Additional file 1: Figure S2B) are regulated by 10 of the 18 TFs (Cebpa/d and Irf8 EU TFs; and Cebpb, Jun, Rela, Spi1, Stat3, Nfkbia, and Atf2 LU TFs). The processes represented by EU and/or LU genes (Groups 2 and 3 in Additional file 1: Figure S2B) are regulated by most of the 18 TFs. The processes represented by the DN genes (Group 4 in Additional file 1: Figure S2B) are regulated mainly by eight of the 18 TFs (Jun, Rela, Atf2, Cebpb, Stat3, Spi1, and Srebf1 LU TFs; and Sox2 DN TF). Taken together, the TRN delineated by the 18 key TFs and the 315 target genes effectively covered transcriptional regulation of early and late cellular processes associated with the pathological features of prion diseases (Fig. 2f).

### Over-represented regulatory motifs in the prion disease-associated TRN

**Fig. 3:**
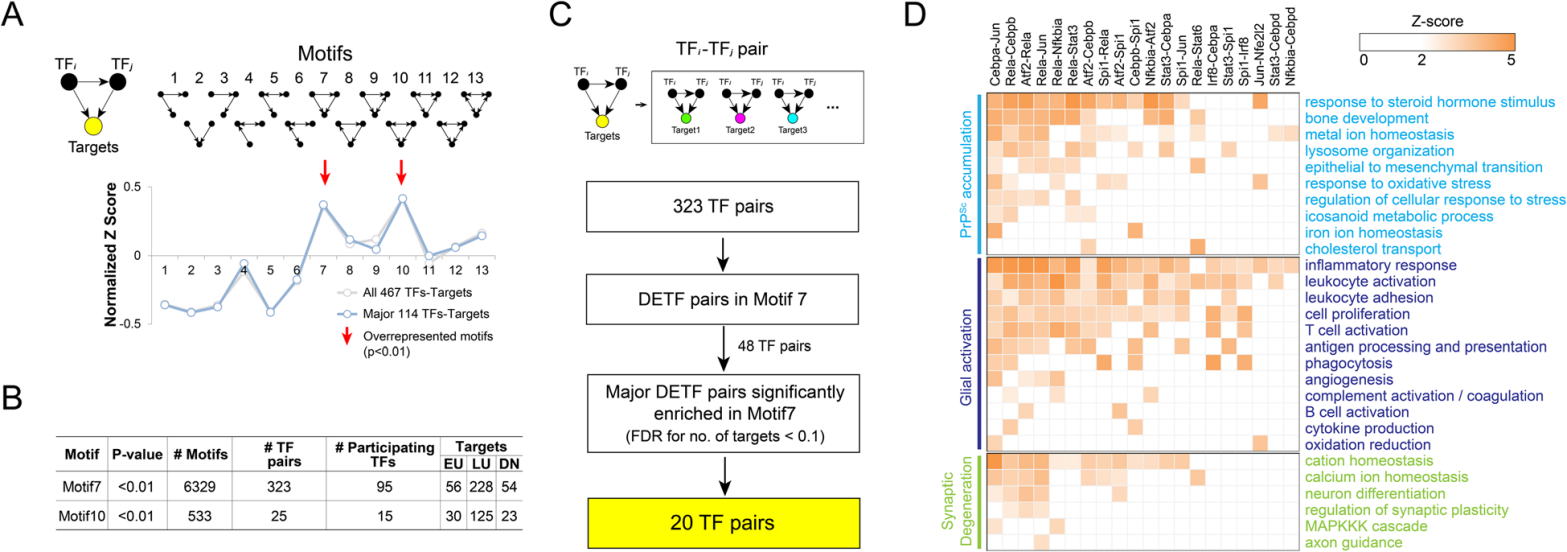
Over-represented regulatory motifs in the TRN. **A**) Two over-represented transcriptional regulatory motifs among the 13 previously reported regulatory motifs. A regulatory structure among two TFs and their targets in each motif is shown (top left). A large positive Z-score indicates significant over-representation of the corresponding motif. Two (Motifs 7 and 10) significantly (*P* < 0.01) over-represented motifs were denoted by the arrows. **B**) Regulatory information of the two over-represented motifs, Motifs 7 and 10. The over-representation p-value, the number of the motifs in the TRN, the numbers of TFs and TF pairs in the motifs, and the numbers of EU, LU, and DN targets in the motifs are shown. **C**) A schematic for identification of key DETF pairs significantly enriched in Motif 7. See text for details. **D**) Heat map showing prion disease-associated processes significantly targeted by the key DETF pairs. Statistical significance (*P*-value) was computed with the hypergeometric test and then transformed into Z-score (Materials and Methods), which was displayed in the heat map

Several methods have been developed for analyzing regulatory structures of biological networks to identify core regulatory motifs or modules in the networks, including significant area search, network propagation, clustering-based methods, and network motif analysis [6]. To determine a method suitable for our TRN, we first examined the topological characteristics of the original TRN constructed for all 467 TFs by analyzing distributions of degrees and clustering coefficients for nodes. The distributions showed that the TRN has the features of both scale-free and hierarchical networks (Figs. S3A-B), indicating that the TRN may include functional regulatory motifs [33]. Collective operations of network motifs composed of TFs and their targets characterize early and late induction or repression of target genes. Thus, among the previous methods, we employed the analysis of network motifs (Fig. 3a, top left) to examine detailed transcriptional regulatory structures in our TRN associated with early and late alterations of target genes.

**Fig. 4:**
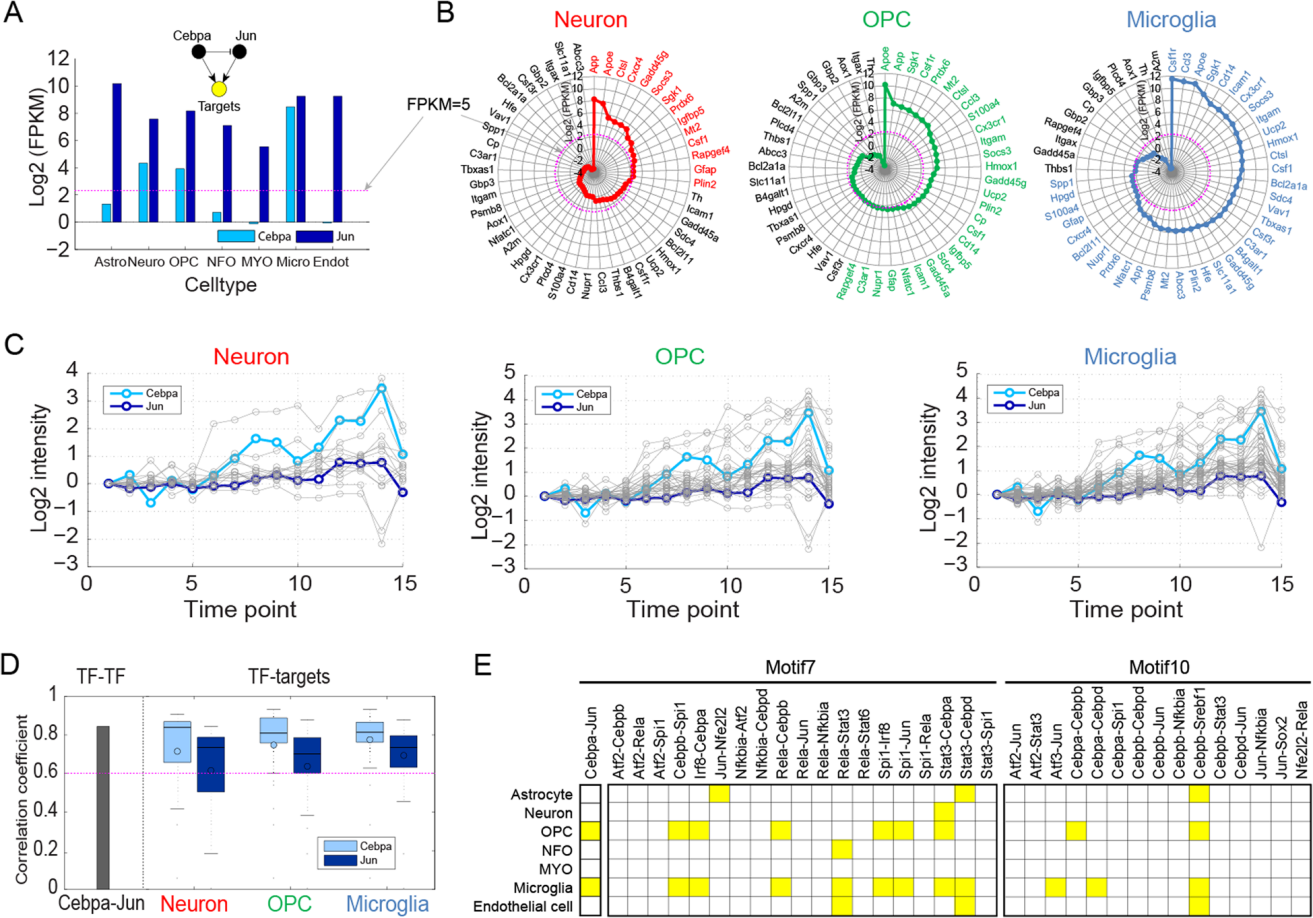
Regulatory motifs potentially operative in individual cells of the brain. **A**) Expression levels (FPKMs) of *Cebpa* and *Jun* in astrocytes (Astro), neurons (Neuro), oligodendrocyte precursor cells (OPCs), newly formed oligodendrocytes (NFOs), myelinating oligodendrocytes (MYOs), microglia (Micro), and endothelial cells (Endot). Colors indicate *Cebpa* (sky blue) and *Jun* (dark blue), respectively. **B**) Radar plots showing expression levels (FPKMs) of target genes regulated by Cebpa-Jun pair in the indicated cells. Different colors were used to distinguish the cell types. **C**) Log_2_-fold-changes of target genes regulated by the Cebpa*-*Jun pair in the B6-RML combination. Colors indicate *Cebpa* (sky blue), *Jun* (dark blue), and targets (gray). **D**) Pearson correlation coefficients between log_2_-fold-changes of *Cebpa* and *Jun* (left bar) and distributions of Pearson correlation coefficients of log_2_-fold-changes of their target genes with those of *Cebpa* (sky blue boxplot) or *Jun* (dark blue boxplot). **F**) Core TF pairs that could be operative in each cell type, which were highlighted in yellow

For network motif analysis, we performed the enrichment analysis of 13 different previously reported regulatory motifs, each of which included a pair of TFs and a target gene (TF_*i*_-TF_*j*_ in Fig. 3a, upper left), as previously described [47]. In this analysis, we used two TRNs constructed for all 467 TFs in addition to the 114 major TFs, not for the TRN for the 18 key DETFs to avoid bias toward the key DETFs in the enrichment analysis. The analysis revealed that among the 13 regulatory motifs, only two motifs (Motifs 7 and 10) including feed-forward loops were significantly (*P* < 0.01) over-represented consistently in the two TRNs (Fig. 3a). Thus, we used the TRN for the 114 major TFs for the following analysis to focus on Motifs 7 and 10 defined by the major TFs. The TRN included 6329 Motif 7 and 533 Motif 10 that comprised 95 and 15 major TFs, respectively, and large portions of the DEGs as target genes (56 EU, 228 LU, and 54 DN genes for Motif 7; and 30 EU, 125 LU, and 23 DN genes for Motif 10) (Fig. 4b**;** Additional file 2: Table S5). Interestingly, Motifs 7 and 10 were also found to be over-represented in the TRNs for *E. coli*, yeast, fly, and/or sea urchin [47], suggesting that the TRN in prion disease includes key regulatory structures conserved in mammalian TRNs in spite of the incomplete nature of TF-target data.

We showed above that the DETFs have higher contributions to regulation of target genes than non-DETFs (Figs. 2a-c). Thus, among the TF pairs in Motifs 7 and 10, we next selected 48 and 20 DETF pairs from 6329 Motif 7 and 533 Motif 10, respectively (Fig. 3c and Additional file 1: Figure S3C**,** 2nd box). Finally, among them, we identified 20 and 15 key DETF pairs that were significantly (FDR < 0.1) enriched in the 6329 Motif 7 and 533 Motif 10, respectively (Fig. 3c and Additional file 1: Figure S3C, 4th box). Interestingly, these selected key DETF pairs (Fig. 3d) included the 18 key DETFs (Fig. 2d), targeting different sets of 178 genes in Motifs 7 and 10, respectively. To understand the contribution of the key DETF pairs to the regulation of cellular processes related to prion disease, we then examined how significantly their target genes in Motifs 7 and 10 overlapped with the DEGs involved in cellular processes (Fig. 1d) associated with the pathological features in prion diseases. A majority of the key DETF pairs showed significant (*P* < 0.01) overlaps of their targets with the DEGs involved in cellular processes related to PrP^Sc^ replication and accumulation and microglia and astrocyte activation, suggesting their strong associations with these pathological features (Fig. 3d and Additional file 1: Figure S3D).

### Regulatory motifs that can be operative in individual cells of the brain

In diverse types of cells, such as astrocytes, microglia, oligodendrocytes, and neurons in the brain, molecular networks may undergo alterations during the progression of prion disease [7]. Our gene expression data were generated from the whole brain, providing mRNA expression levels from the mixture of diverse cells in the brain. Thus, it is difficult to sort out in what cell types Motifs 7 and 10 were altered by prion infection. To examine whether Motifs 7 and 10 can be operative in individual cell types, we first analyzed whether both TFs in each selected TF pair were expressed in the same cell type using previously reported gene expression data of seven cell types in the mouse brain, including microglia, astrocytes, neurons, oligodendrocyte precursor cells (OPCs), newly formed oligodendrocytes (NFOs), myelinating oligodendrocytes (MYOs), and endothelial cells [48]. For example, for Cebpa-Jun pair forming Motif 7, both *Cebpa* and *Jun* were found to be expressed only in microglia, neurons, and OPCs among the seven cell types (Fig. 4a). To evaluate whether TFs were expressed, FPKM ≥5 was used as a cutoff as in Zhang et al [48]

We then examined whether such expressed TF pairs had significant regulatory power by analyzing the numbers of their target genes expressed in the same cell types. The Cebpa-Jun pair had 51 targets in the 6329 Motif 7, and significant (FDR < 0.1) numbers of the target genes were found in all three cell types (14, 28, and 37 genes in neuron, OPCs, and microglia, respectively) (Fig. 4b), suggesting that Motif 7 formed by Cebpa-Jun pair and their targets can be operative with significant regulatory power in these three cell types. Finally, we analyzed the correlation between differential expression patterns of TFs and their targets, which were expressed in the same cell type, in the whole brain data for the B6-RML combination along disease progression (Fig. 4c). For the Cebpa-Jun pair, *Cebpa* and *Jun* first showed a significant (*P* < 0.01) positive correlation (0.84) between their differential expression patterns along the progression of prion disease (Fig. 4d). Additionally, the targets expressed in OPCs and microglia, yet not in neurons, showed significant positive correlations (≥ 0.6 statistically) with *Cebpa* and *Jun* (Fig. 4d). All these data suggest that Motif 7 formed by Cebpa-Jun and its targets can act as a functional motif with significant regulatory power in OPCs and microglia during the progression of prion disease.

We applied the same procedure to all 20 and 15 key DETF pairs selected for Motifs 7 and 10, respectively, and identified 8 and 6 key DETF pairs for Motifs 7 and 10, respectively, which could be operative with significant regulatory power in one of the seven cell types (Fig. 4e and Additional file 1: Figure S4). Interestingly, these DETF pairs were found to have significant regulatory power most strongly in microglia (9 and 3 pairs for Motifs 7 and 10, respectively) and OPCs (7 and 2 pairs for Motifs 7 and 10, respectively). Only few DETF pairs had significant regulatory power in astrocytes (3 pairs), neurons (1 pair), MYO (0 pair), and endothelial cells (3 pairs). Moreover, the comparison of the DETF pairs between microglia and OPCs revealed that the target gene counts for the DETF pairs were higher in microglia than in OPCs (Fig. 4b). Collectively, all these data suggest that these key regulatory motifs are employed in microglia and OPCs to regulate the target genes in the two cell types. Moreover, they are more strongly operative in microglia than in OPCs.

### Core transcriptional regulatory circuits for dynamic activation of cellular processes

**Fig. 5:**
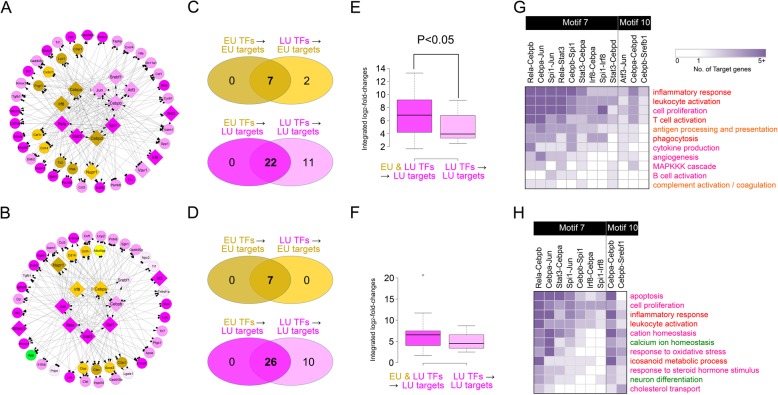
Core transcriptional regulatory circuits (TRCs) in microglia and OPCs and characteristics of TRCs. **A-B**) TRCs in microglia (**A**) and OPCs (**B**). Node shapes represent TFs (diamonds) and target genes (circles), and large diamonds denote DETFs. Node colors represent EU (orange), LU (magenta), and DN (green) genes. Edge types indicate activation (arrow), inhibition, and unspecified interactions (blunted arrow). **C-D**) Relationships among EU (top Venn diagram) and LU (down Venn diagram) target genes regulated by EU or LU TFs in microglial (**C**) and OPC (**D**) TRCs. Label colors indicate EU (orange) and LU (magenta) genes. **E-F**) Comparison of integrated log_2_-fold-changes of LU target genes regulated by EU and LU TFs in microglial (**E**) and OPC (**F**) TRCs. P-value was computed by two-tailed Student t-test. **G-H**) Heat map showing the numbers of target genes included in microglial (**G**) and OPC (**H**) TRCs. Numbers of target genes involved in prion disease-associated GOBP were counted and displayed in the heat map

To understand collective actions of the regulatory motifs, we next combined all the regulatory Motifs 7 and 10 that could be operative in microglia and OPCs with strong regulatory power and identified core transcriptional regulatory circuits (TRCs) including key DETF pairs and their target genes in the selected Motifs 7 and 10 in the two cell types (Figs. 5a-b). In the microglial and OPC TRCs, EU and LU genes were targeted by EU or LU DETFs and found to be more regulated by both EU and LU DETFs than by either EU or LU DETFs alone (Figs. 5c-d). Interestingly, for the LU target genes, their fold-changes tended to be higher when regulated by both EU and LU DETFs than by either EU or LU DETFs alone (Figs. 5e-f). All these data suggest that EU DETFs modulate the expression of LU target genes, and LU DETFs also modulate the expression of EU target genes at the late stage of prion diseases.

Comparisons of the nodes in the microglial and OPC TRCs showed that 8 DETFs and 18 target genes were shared between the two TRCs, which corresponded to 80% of the DETFs and 43% of the target genes in the microglial TRC, suggesting that the same DETFs target different sets of genes between microglia and OPCs (Figs. 5a-b). The target genes in the microglial TRC were mainly involved in early inflammatory and immune responses (Fig. 1d) represented by the EU genes (Fig. 5g**;** Additional file 2: Table S6). Compared to the microglial TRC, the OPC TRC included the target genes that were mainly involved in the relatively later immune responses and responses to oxidative stress and steroid hormones represented by the LU genes, as well as calcium homeostasis and neuron differentiation represented by the DN genes (Fig. 5h**;** Additional file 2: Table S6). Moreover, we compared the nodes in our TRCs with cell type specific DEGs between no pathology and AD pathology obtained from single cell RNA sequencing of prefrontal cortex samples from 24 control individuals with no or little pathology (no-pathology) and 24 age-matched individuals with a spectrum of mild to severe β-amyloid and other pathologies (AD-pathology) [49]. The comparison revealed that Spi1 and Tnfrsf1 in the microglial TRC were shared with microglia specific DEGs identified from single cell RNA sequencing and Gadd45 in the OPC TRC was with OPC specific DEGs. Taken together, these data suggest that TRCs represent core regulatory programs underlying early and late alterations of cellular processes associated with prion disease.

## Discussion

Perturbation of biological networks leads to activation of a series of pathophysiological processes at early and late stages during the course of disease progression. Transcriptional regulation is a primary control mechanism underlying temporal activation of the pathophysiological processes. Prion diseases induce early and late expression changes of hundreds of genes, leading to alteration of cellular processes associated with PrP^Sc^ accumulation, microglial/astrocytic activation, synaptic degeneration, and neuronal cell death. Thus, decoding TRNs that describe transcriptional regulation associated with early and late activation of cellular processes during disease progression is essential to understanding the pathogenesis of prion diseases. However, the dynamics of TRNs in prion diseases had not yet been systematically explored. In this study, we present a TRN that contains two over-represented feed-forward loops (regulatory Motifs 7 and 10), which can serve as the basis for transcriptional regulation of target genes. Therefore, our TRN provides transcriptional regulatory programs representing early and late alteration of target gene expression and their associated processes during the progression of prion diseases.

Due to the incomplete nature of the TF-target interactome (and focusing on over-presented TF-pairs), our TRN provides only a partial view of transcriptional regulation in prion diseases. Some known TFs involved in the pathogenesis of prion diseases, such as Notch contributing to atrophy of dendrites in prion diseases [23], were not included in the 18 key TFs used for the TRN. Thus, the list of the regulatory motifs and those motifs that are over-represented in our TRN may be incomplete. ChIP-chip or seq analysis of the known TFs and the DETFs can provide further assessment of transcriptional regulation underlying expression changes of the genes associated with the pathophysiological features in prion diseases. Nevertheless, the over-represented regulatory motifs in our TRN provide an informative transcriptional regulatory framework for early and late alteration of cellular processes during disease progression. Over-representation of Motifs 7 and 10, consistent with those in the TRNs of yeast and *E. coli* with more comprehensive TF-target interactomes, support the utility of the over-represented regulatory motifs in our TRN.

It was previously demonstrated that the feed-forward loops in Motifs 7 and 10 can cause delays (coherent feed-forward loops) or accelerations (incoherent feed-forward loops) in induction of target genes when TF pairs are activated [2, 6]. Thus, such accelerated and delayed induction of target genes caused by the feed-forward loops can be speculated to contribute to early (EU genes) and late (LU and DN genes) alterations of the target genes. However, the time interval in our time-course gene expression profiling was 2 weeks for BL6-RML and FVB-RML and 4 weeks for Prnp0/wt-RML. Given these time intervals, considering that transcriptional induction of target genes in the feed-forward loops occurs within several hours, the effects of the feed-forward loops on the induction kinetics of target genes may not be apparent in our time-course gene expression data. Therefore, we considered the over-represented motifs as the feed-forward loops that represent early and late alterations of target genes in the TRN during disease progression.

Gene expression profiles used in this study were generated from the whole brain. Dynamic signatures of mRNA expression observed at the whole brain level can be different at the cellular level because of the mixing of mRNA expression signatures across various types of cells affected by PrP^Sc^. Thus, the validity of dynamic expression patterns of mRNA signatures in inferring the over-represented regulatory motifs that are operative in individual cells are those of importance. To address this issue, we integrated mRNA expression signatures in seven types of cells in the brain with those in the whole brain and further selected Motifs 7 and 10 where TF pairs and their target genes that were expressed in the same cell type and their expression patterns in the whole brain were significantly correlated. The selected Motifs 7 and 10 were then considered operative in the cell types. The integration of mRNA expression signatures at both organellar and cellular levels might be an effective way that can resolve the intrinsic issues of the individual organ- and cell-level data.

Microglial and OPC TRCs shared 8 DETFs (Irf8, Cebpa, Jun, Rela, Stat3, Spi1, Cebpb, and Srebf1) and 18 target genes (Ccl3, Cd14, Csf1, Csf1r, C3ar1, Nupr1, Hmox1, Icam1, Itgam, Blnk, Gadd45g, Il10rb, Lyn, Tlr7, Tgfb1, Trf, Tnfrsf1, and Dab2) that appear to be more relevant to prion diseases than other genes in the TRCs. Previous studies have shown potential associations of these TFs and target genes with prion diseases or other neurodegenerative disorders. Among the shared TFs, Cebpa is required for differentiation of myeloid and Cebpa expression is induced in prion diseases [2, 38] and its expression correlates with clinical scores of incipient AD [50]. A recent study shows that Cebpa ameliorates dendritic abnormalities induced by activation of MT2 receptor together with miR125b in AD [51]. Irf8 is a critical TF for microglial activation and promotes neuroinflammation under neurodegenerative conditions of AD and EAE, a mouse model for multiple sclerosis [52–54]. Spi1, also known as PU.1 in humans, is a master regulator of microglial gene expression and reductions in Spi1 delayed disease development of AD [55]. Spi1 and Irf8 cooperatively regulate microglial activation in neurodegenerative conditions [53, 56], consistent with a Motif 7 regulatory relationship including Spi1 and Irf8 in the TRCs. Phosphorylation of Jun plays key roles during early phase of neuronal death induced by prions [45, 57]. Central nervous system-specific deletion of Rela accelerates prion disease via increased neuronal cell death [58]. Jak-Stat signaling, including Stat3, is up-regulated in prion, Alzheimer’s, and Huntington’s diseases [43, 59], and promotes astrogliosis in scrapie-infected mice [60]. Cebpb regulates expression of delta secretase to modify amyloid plaque formation in AD [61] and promotes glial activation in Parkinson’s disease models [62]. Srebf1 links lipogenesis to mitophagy and sporadic Parkinson’s disease [63], and knockdown of Srebf1 blocks the translocation of Parkin into mitochondria, thereby decreasing mitophagy [64].

Among the shared target genes, Cd14 is a glycosylphosphatidylinositol-anchored receptor known as a co-receptor for TLRs [65] and critical for Tlr2-mediated macrophage activation [66]. Absence of Cd14 delays progression of prion diseases accompanied by increased microglial activation [67]. Inhibition of Csf1r decreases microglial proliferation and delays neuronal damage in prion disease [56]. Csf1r is activated together with Cebpa and Spi1 in prion diseases, consistent with a Motif 10 regulatory relationship including Cebpa-Spi1 and Csfr1 in the TRCs. C3ar1 inactivation attenuates Tau pathology by reversing deregulated immune networks in Tauopathy models and AD [68]. Nupr1 is emerged as an important TF in the growth and migration of human glioblastoma cells [69] and a potent regulator of autolysosomal dynamics via the induction of the SNARE proteins. Nupr1 depletion impairs autolysosomal clearance and induces cytoplasmic vacuolization, suggesting a key role in neuronal autophagy [70, 71]. Ccl3 is a known activator of Jak-Stat signaling during prion-induced gliosis [43, 72], and deletion of Tnfrsf1 reduces the number of amyloid plaques and cognitive deficits in AD mouse models [73]. Icam1 and Itgam are up-regulated during scrapie infection [72, 74], and Icam1 is known to be regulated cooperatively by Nfkb and Stat signaling [75], consistent with a Motif 7 regulatory relationship including Rela-Stat3 and Icam1. Unlike the above inflammatory genes that promotes disease progression, several inflammation-related target genes have neuroprotective functions. Deficiency of Tgfb1 signaling increases both Aβ accumulation and Aβ-induced neurodegeneration in AD models [76], and absence of Il10 accelerates prion disease [77], suggesting neuroprotective roles of Il10-Il10rb signaling. Additionally, Dab2 attenuates brain injury in AD mouse models via targeting Tgfb1 signaling [78]. Overexpression of Hmox1 contributes to mitochondrial damage in AD and Parkinson’s disease models [79]. Iron homeostasis is regulated by Trf, which increases with PrP^Sc^ levels, accounting for prion-disease associated iron deficiency in scrapie-infected mouse and hamster brains [80]. Taken together, these and other functional associations of the shared TFs and their target genes with neurodegenerative diseases support the validity of our TRCs.

A panel of Motifs 7 and 10 that could be operative in a cell type act together for coordinated regulation of the expression of target genes in the corresponding cell. To understand the collective action of Motifs 7 and 10, we reconstructed TRCs for microglia and OPCs. However, the target genes in the TRCs were regulated by multiple TFs in complicated ways, making it difficult to clearly interpret transcriptional regulatory relationships between TFs and target genes. Nevertheless, the most apparent observation of the TRCs in operation was that both EU and LU DETFs could regulate EU or LU target genes, modulating the expression of EU or LU target genes at the late stage during the course of disease progression. Detailed functional studies can be designed to investigate regulatory roles of EU or LU DETFs in regulation of EU and LU target genes and how such regulations contribute to PrP^Sc^ replication and accumulation, synaptic degeneration, and neuronal cell death**.**

The disease-perturbed networks become more complex as disease progresses. This is why earliest possible diagnosis (biomarkers) and therapy (drug targets) are essential for treating diseases. In this study, we decoded the dynamic transition of the TRN describing the major pathophysiological features of prion disease and then identified the core transcriptional regulatory circuits in prion disease through regulatory motifs and cell type analysis. Importantly, the core circuit demonstrates the transcriptional regulatory pathways important for microglial activation, an important early pathophysiological feature in prion disease. The EU-DETFs (Irf8, Cebpa, Cebpd, Nupr1) in the early regulatory pathways can serve as biomarkers and therapeutic targets for early diagnosis and therapy, respectively. As a result, our systems approach to decipher the dynamics of TRN coupled with motif and cell type analyses provides specific molecular targets for early diagnosis and therapy.

## Additional files

**Additional file 1:** **Figure S1.** Twenty major differential expression patterns identified by orthogonal non-negative matrix factorization (ONMF). **Figure S2.** The transcriptional regulatory network (TRN) describing the regulation of target genes by 467 TFs. **Figure S3.** The topological characteristics of the TRN and over-represented Motif 10. **Figure S4.** Selected key DETF pairs for Motifs 7 and 10.

**Additional file 2:** **Table S1.** Previously reported differential expression of 883 genes during the progression of prion diseases. **Table S2.** GO Biological Processes enriched by EU, LU, and DN genes. **Table S3.** 114 major TFs and previously reported associations with prion or other neurodegenerative diseases. **Table S4.** Representative cellular processes associated with prion diseases. **Table S5.** Motif7 - 6329 subnetworks and Motif10 - 533 subnetworks. **Table S6.** GOBPs regulated by TF pairs that could be operative in microglia and OPC.

## Data Availability

The dataset supporting the conclusions of this article is available in the ArrayExpress (E-MTAB-76, https://www.ebi.ac.uk/arrayexpress/experiments/E-MTAB-76/?query=prion+mouse&page=1&pagesize=50). Expression dataset that had been used to analyze brain cell specific genes can be found at GSE52564.
