# Supplementary material for: Core transcriptional regulatory circuits in prion diseases
Source: Mol Brain. 2020 Jan 20;13:10. doi: 10.1186/s13041-020-0551-3 (PMC6972013; doi:10.1186/s13041-020-0551-3)
Supplement: Supplementary file 1 — Additional file 1: Figure S1. Twenty major differential expression patterns identified by orthogonal non-negative matrix factorization (ONMF). Figure S2. The transcriptional regulatory network (TRN) describing the regulation of target genes by 467 TFs. Figure S3. The topological characteristics of the TRN and over-represented Motif 10. Figure S4. Selected key DETF pairs for Motifs 7 and 10. [file 13041_2020_551_MOESM1_ESM.docx]

**Supplementary Information**

**Core transcriptional regulatory circuits in prion diseases**

Taek-Kyun Kim, Inyoul Lee, Ji-Hoon Cho, Brenda Canine, Andrew Keller, Nathan D. Price, Daehee Hwang, George Carlson, and Leroy Hood


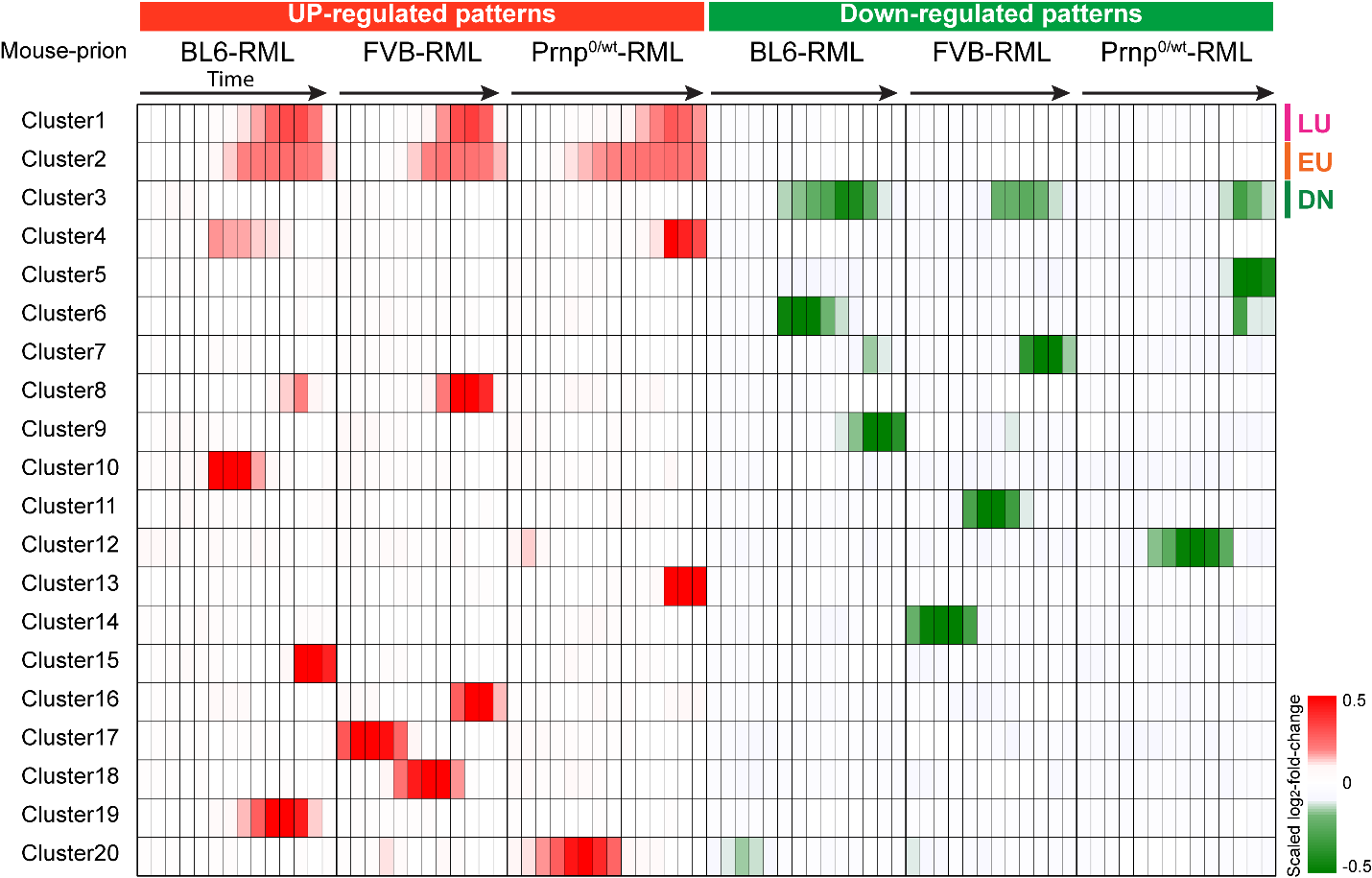


**Figure S1.** **Twenty major differential expression patterns identified by orthogonal non-negative matrix factorization (ONMF).**

Each differential expression pattern (each raw in the heat map) represents the averaged log_2_-fold changes of the genes belonging to each pattern in the three datasets (BL6-RML, FVB-RML, and Prnp^0/+^-RML). The arrow on the top of the heat map denotes the increasing time after prion infection in each dataset. For each pattern, positive log_2_-fold-changes were shown as up-regulation (red) over time in the left half of the heat map while negative log_2_-fold-changes were as down-regulation (green) in the right half of the heat map. The color bar represents the gradient of log_2_-fold changes. According to the significance measures estimated by ONMF, we selected the first three patterns, which represent LU, EU, and DN, respectively, as the most significant differential expression patterns for reconstruction of a transcriptional regulatory network (TRN).


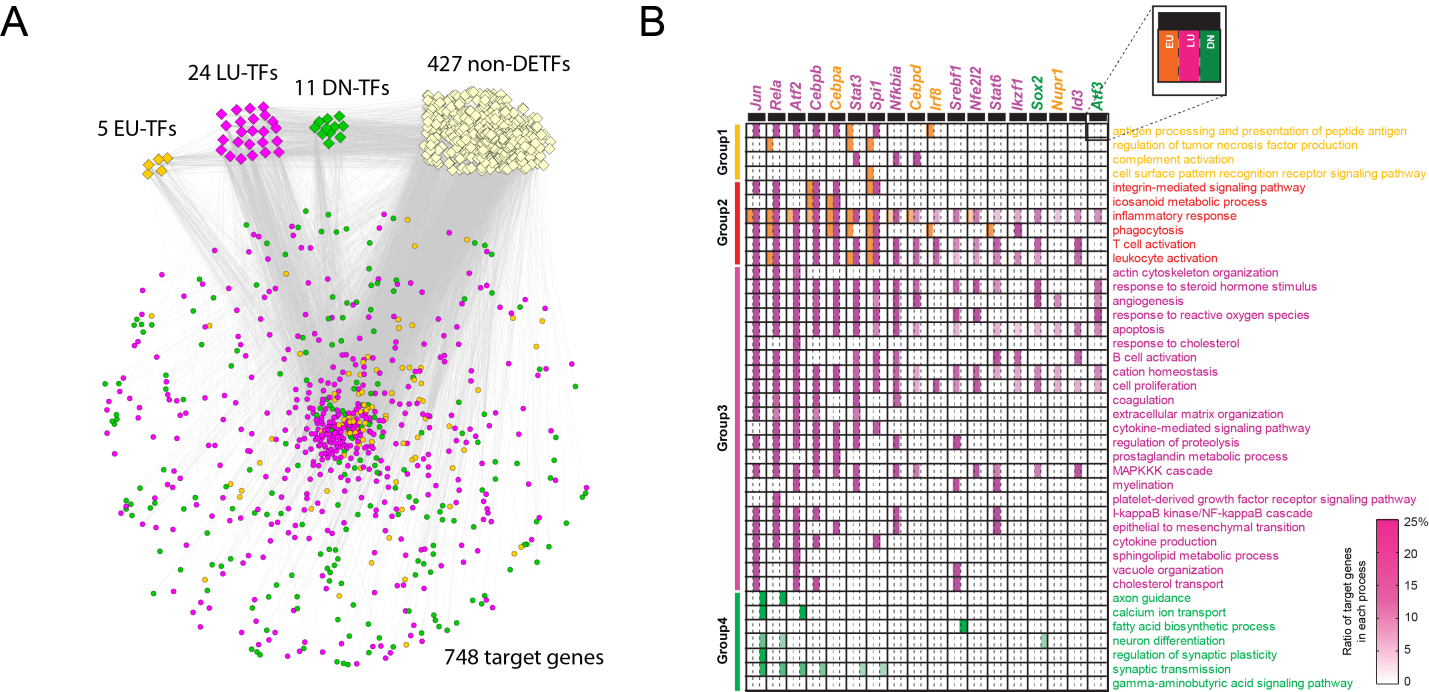


**Figure S2. The transcriptional regulatory network (TRN) describing the regulation of target genes by 467 TFs.**

A) Using the interactions between the 467 TFs and target genes, a TRN was built. The TFs (diamond) and targets (circle) are located on the top and bottom, respectively. Dynamic expression patterns are represented by the node colors (orange for EU, magenta for LU, and green for DN). B) The tabular heat map shows the relationships of 1) TFs, 2) target genes in EU, LU, and DN clusters, and 3) cellular processes associated with pathological features of prion disease (PrP^Sc^ accumulation, microglial/astrocytic activation, and synaptic degeneration in **Figure 1D**). The relationship can be interpreted as follows. First, each of the 18 key TFs (a column in the heat map) regulates EU, LU, and DN target genes (three small boxes in the column; see the legend for the boxes in the top right). The color gradient (see color bar) in the three small boxes represents the percentage of the genes regulated by the TFs among the EU, LU, and DN genes involved in a particular GOBP (each row), respectively. Different colors were used to distinguish EU (orange), LU (magenta), and DN (green). For example, Spi1 regulates mainly LU genes (1^st^ box among the three small boxes) that are involved in antigen processing and presentation of peptide antigen (1^st^ GOBP in the right). The GOBPs were categorized into Groups 1 to 4, according to their involvement in PrP^Sc^ accumulation, microglial/astrocytic activation, and synaptic degeneration (left side of the tabular heat map) as described in **Figure 1D**.


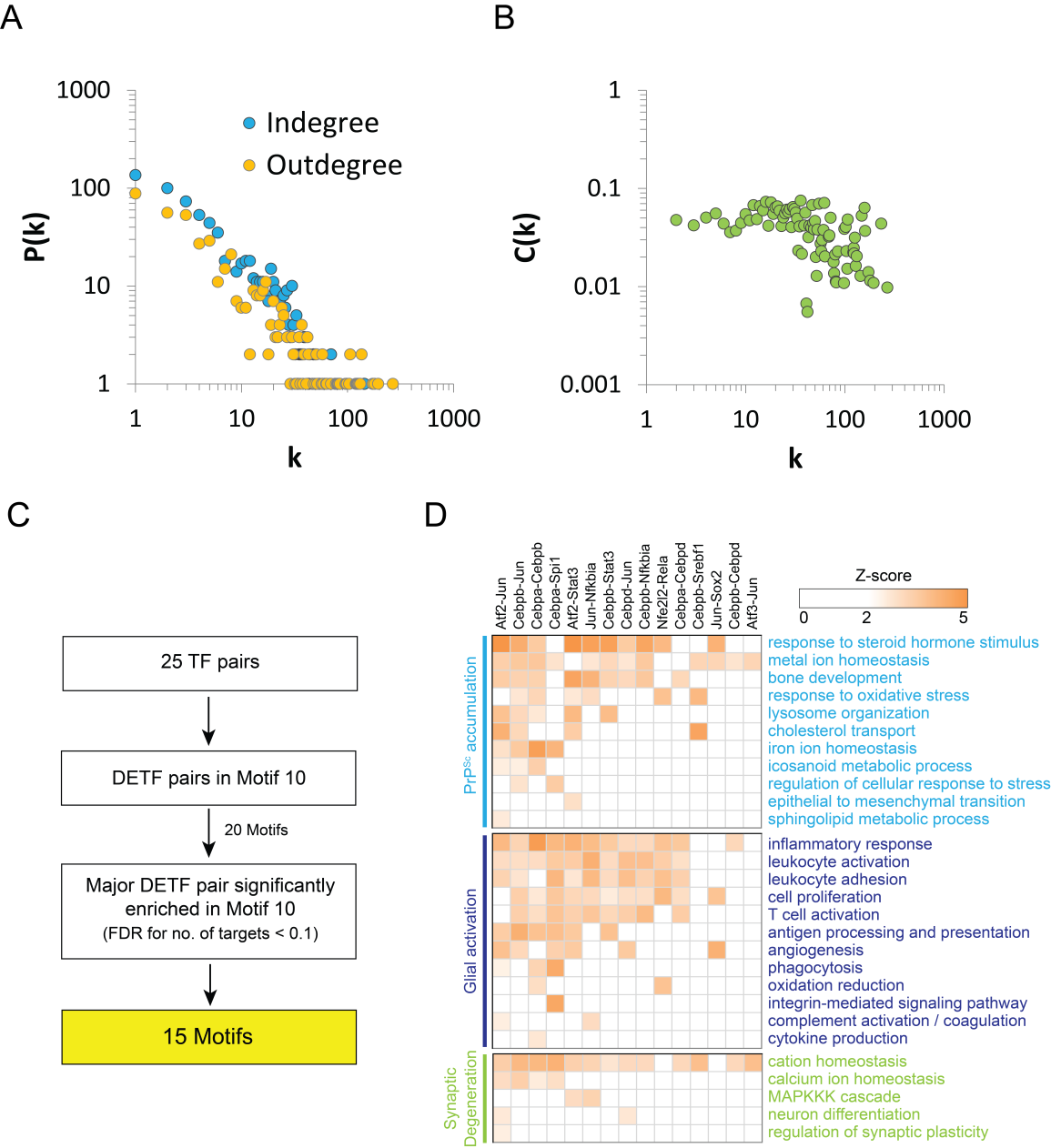


**Figure S3.** **The topological characteristics of the TRN and over-represented Motif 10.**

A-B) Characteristics of scale-free and hierarchical networks. For each of the TFs in the TRN, we computed degree *k* (number of target genes), degree distribution P(*k*) (the number of TFs with degree *k*), and clustering coefficient C(*k*). The relationships between *k* and P(*k*) (A) and between *k* and C(*k*) (B) were shown using log-log scatter plots. The linear relationships on the scatter plots indicate that the TRN has the nature of both scale-free (A) and hierarchical networks (B). C) Schematics for identification of TF pairs that were significantly enriched in 533 Motif 10. First, among TF pairs in 533 Motif 10, 20 DETF pairs were selected. Second, 15 DETF pairs significantly (FDR <0.1) enriched in 533 Motif 10 with significant numbers of targets were selected. D) The heat map showing the overlaps of targets of the 15 DETF pairs with DEGs involved in processes related to pathological features in prion diseases. Statistical significance of the numbers of the overlapped targets of each TF pair was computed with Fisher’s exact test. The p-values were then transformed into Z score and displayed in the heat map. The color bar shows the gradient of the Z score.


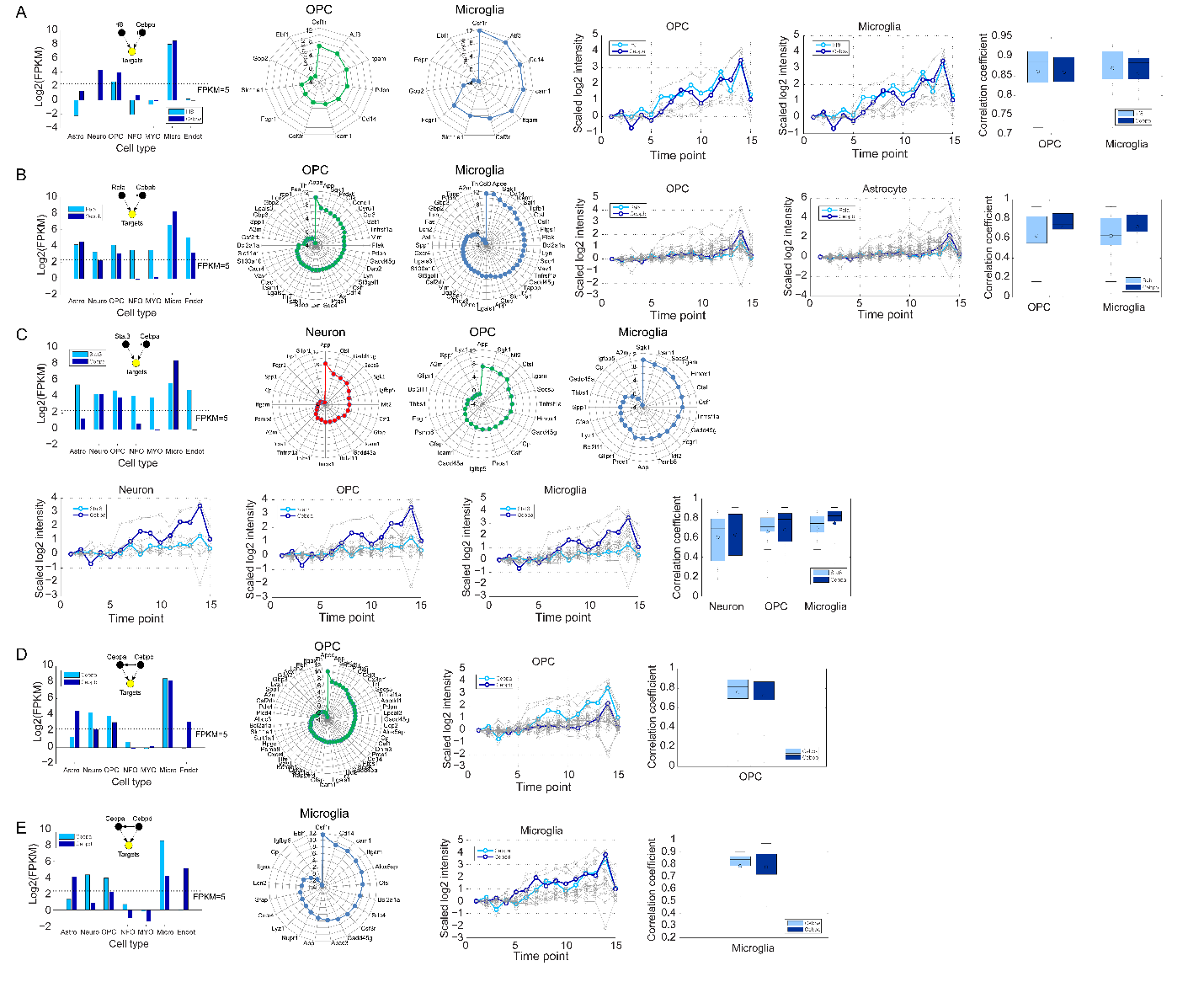


**Figure S4. Selected key DETF pairs for Motifs 7 and 10.**

Expression of TFs in the indicated TF pair across seven cell types [microglia, astrocytes, neuron, oligodendrocyte precursor cells (OPCs), newly formed oligodendrocytes (NFOs), myelinating oligodendrocytes (MYOs), and endothelial cells], target gene expression in the indicated cell type, correlation of TF expression levels measured from the whole brain with those of target genes expressed in the indicated cell type, and distributions of the TF-target gene correlations for three and two selected DETF pairs from Motifs 7 and 10, respectively: Irf8-Cebpa (**A**), Rela-Cebpb (**B**), and Stat3-Cebpa (**C**) from Motif 7 and Cebpa-Cebpb (**D**) and Cebpa-Cebpd (**E**).
